# Transumbilical Laparo-Assisted Appendectomy: A Safe Operation for the Whole Spectrum of Appendicitis in Children—A Single-Centre Experience

**DOI:** 10.1155/2013/216416

**Published:** 2013-03-27

**Authors:** D. Codrich, M. G. Scarpa, M. A. Lembo, F. Pederiva, D. Olenik, F. Gobbo, A. Giannotta, S. Cherti, J. Schleef

**Affiliations:** ^1^Unit of Pediatric Surgery and Urology, Institute for Maternal and Child Health, IRCCS Burlo Garofolo, Trieste, Italy; ^2^Department of Pediatric Surgery, Children's Hospital Burlo Garofolo, Via dell'Istria 65/1, 34100 Trieste, Italy; ^3^Operatory Theatres, Institute for Maternal and Child Health, IRCCS Burlo Garofolo, Trieste, Italy

## Abstract

The paper reports the results of a retrospective review of the medical charts of 203 patients admitted to a pediatric surgical unit with a diagnosis of acute appendicitis between January 2006 and December 2010 when a transumbilical laparoscopic-assisted appendectomy (TULAA) was introduced as a new surgical technique. Among 203 admitted patients, 7 (3.5%) had a localized appendiceal abscess and were treated with antibiotics. All of them responded to antibiotics and underwent TULAA interval appendectomy 8 weeks later. 196 patients (96.5%) underwent immediate surgery. In 12/181 (6.6%) urgent cases, conversion to laparotomy was necessary, in 3 patients because of bowel distension and in 9 for retrocecal position of appendix. In all 181 TULAA completed procedures, one trocar was used in 151 cases (89.4%), two trocars in 16 (9.4%), and three trocars in 2 (1.2%). The mean operative time for single port TULAA was 52′ Complications included 5 wound infections and 5 intra-abdominal abscesses, all managed conservatively. In conclusion, TULAA is a safe, minimally invasive approach with acute appendicitis, regardless of the perforation status, and can be recommended in the pediatric urgical settings.

## 1. Introduction

Appendicitis is the most frequent indication for urgent surgery in children. Since 1894, when Mc Burney described the laparotomic technique for appendectomy, the same operation has been the gold standard for acute appendicitis for over a century. In 1983, Semm [1] described for the first time the standard three ports laparoscopic appendectomy and since then the minimally invasive approach has gained wide acceptance among the pediatric surgeons. Different variations of the laparoscopic technique have been proposed, all aiming to better cosmetic results, reduction in costs, and charges for hospitals, while keeping the safety of the operation unchanged. The umbilicus as the unique site to gain access to the abdomen and to the appendix has been widely reported in the literature, both as a port to exteriorize the appendix and perform an extracorporeal operation [2, 3] and as the site to place all laparoscopic instruments and perform an intracorporeal appendectomy (SILS; single-site laparoscopic surgery) [4, 5]. The trans umbilical laparo-assisted technique (TULAA) merges together the advantages of both a good intraabdominal laparoscopic visualization and the safety and quickness of an extracorporeal traditional appendectomy. A large series of pediatric patients operated on with this technique was presented in 1999 by Valla et al. [2], but patients were selected for absence of complicated appendicitis. Recently, Ohno et al. presented a paper in which the TULAA procedure was used in 416 patients but without any perforated appendicitis or local abscesses in the series [6]. 

We present the experience of our centre, in which the use of TULAA was firstly introduced in 2006, in a team where only one surgeon had used the technique before, and it was decided to perform it with every kind of appendicitis, with or without the suspect of complicated appendicitis. 

## 2. Materials and Methods

The charts of all patients admitted to our surgical department from January 2006 to December 2010, with a diagnosis of appendicitis based on clinical (migration of pain to right lower quadrant (RLQ), fever, and rebound tenderness in RLQ), laboratory (elevated WBC count, elevate C Reactive Protein (CRP)), and ultrasound (US) findings were retrospectively reviewed for demographical data, surgical treatment, time for completing the operation, intraoperative finding, need for conversion, and surgical complications. 

Before 2006, all suspected appendicitis, regardless of history and perforation status, were treated by open surgery, and antibiotic therapy was prescribed according to the preference of the surgeon. Since 2006, a new protocol for the treatment of complicated and uncomplicated appendicitis was introduced in our surgical department.

### 2.1. Protocol of Treatment

All patients with suspected nonperforated or perforated appendicitis but with a history of less than 72 hours and no ultrasound evidence of consolidated appendiceal mass are offered TULAA. All patients undergoing surgery are administered a single dose of ampicillin plus sulbactam (50 mg/kg/dose) as prophylaxis 30′ before starting the operation. If there is no perforation, the therapy with the same antibiotic is continued for 24 hours and then stopped; whenever perforation is found, a regimen of ceftriaxone (100 mg/kg/die in one administration) plus metronidazole (7.5 mg/kg/dose every 8 hrs) is administered as long as the patient is afebrile for at least 24 hours, inflammatory markers are diminishing, and full oral diet is restored. In case of discharge before 7 days of intravenous antibiotics, patients are put on oral amoxicillin (50 mg/kg/dose every 12 hrs) and metronidazole (7.5 mg/kg/dose every 8 hrs) to complete a whole week of therapy. 

All appendiceal masses (symptoms lasting for at least 72 hours before presentation and US confirming the presence of a consolidated appendiceal abscess) are admitted to the ward and treated conservatively with an antibiotic regimen of ampicillin plus sulbactam (50 mg/kg/dose every 8 hrs), metronidazole (7.5 mg/kg/dose every 8 hrs), and tobramicina (5 mg/kg/die in one administration). After 48 hours of antibiotics, the patients are evaluated clinically, and inflammatory markers (CRP and WBC) are repeated: if laboratory and clinical improvements are observed, the antibiotic therapy is continued until the patients are afebrile for at least 48 hours, inflammatory markers are progressively diminishing, and oral diet is resumed. After 8 weeks, an interval TULAA is performed. If no improvements are seen after 48 hours of antibiotics, the patients are offered TULAA. Appendiceal abscesses with US evidence of a fecalith are treated with immediate TULAA since the fecalith is a known risk factor for abscess persistence [7].

Patients are started on a liquid diet 12 hours after the operation and on semiliquid diet in the first postoperative day. Gradually, in 48 hours, full oral diet is restored in uncomplicated cases. Criteria for discharge are patient afebrile for at least 24 hours, restoration of full oral diet, and decreasing inflammatory markers.

### 2.2. Surgical Technique

The patient is placed in the supine position under general anesthesia and mechanical ventilation. No bladder catheterization is used since all patients are asked to void before entering the operatory theatre.

**Figure 1:**
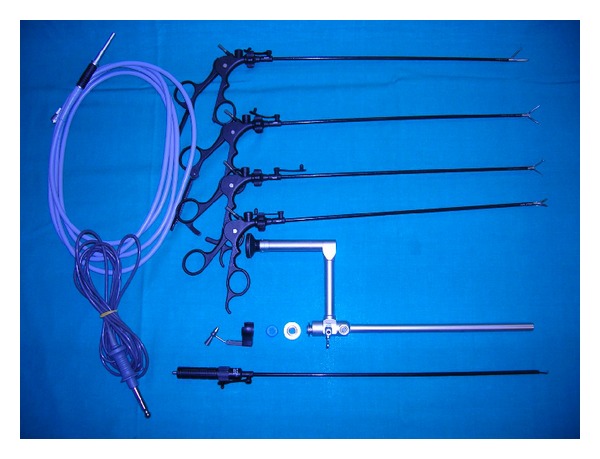
The instrumentation for the TULAA appendectomy: cautery hook, operative scope, and long graspers.

A single-infraumbilical incision is performed, and an 11 mm balloon trocar is inserted under direct visualization. Capnoperitoneum is maintained within a range of 8 to12 mmHg according to the bodyweight of the patient with insufflation of CO_2_ at a rate of 1.5 L/min. A single-operative laparoscope (Karl Storz Endoskope, Hopkins optical devices) with a side-arm viewing is inserted through a single, transumbilical port (Figure 1), and a grasper is used to identify the appendix and to dissect retroperitoneal adhesions: when the tip of the appendix is freed, it is exteriorized through the umbilicus. An extracorporeal appendectomy is performed by dividing and ligating the mesoappendix, suture ligation, and inversion with purse string of the appendiceal base. No endomechanical devises are used. In case of difficult dissection, one or two further additional 5 mm trocars for additional graspers or cautery hook might be introduced.

 At the end of the procedure, intraperitoneal local anesthetic drugs such as naropine 0.2% at a dose of 0.5 mL/kg are instilled in the peritoneal cavity through one of the trocars. Postoperative analgesia is administered via an elastomeric intravenous pump with tramadol 2–8 mcg/kg/min for 24 hours plus repeated doses of paracetamol 10 mg/kg every 8 hours. Nausea is controlled by ondansetron 0.15 mg/kg every 8 hours, and rescue analgesic therapy consists of ketoprofene 1 mg/kg every 8 hours.

When the appendectomy is considered impossible to be safely completed with any laparoscopic technique, it is converted to an open access. 

A primary open access is chosen only when the performing surgeons are not trained in laparoscopy or abdominal distension is prominent.

An expert TULA surgeon is defined as a surgeon who has performed at least 30 procedures as first operator or is trained in laparoscopy.

## 3. Results

**Figure 2:**
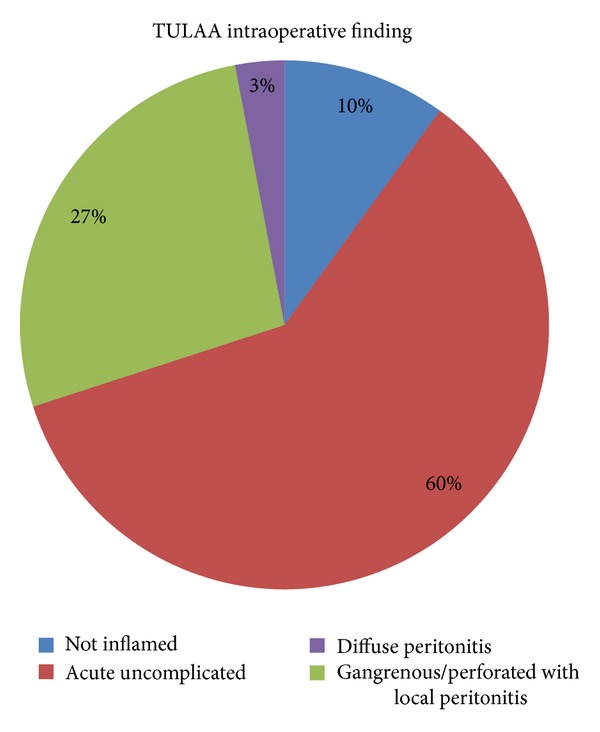
TULAA intraoperative finding. Macroscopic staging of the appendiceal inflammation.

From January 2006 until December 2010, 203 patients (79 female and 124 male) with an average age of 10 years (range 3–17) were admitted to our surgical ward with a diagnosis of appendicitis. Seven (3.4%) out of 203 patients presented with an appendiceal mass and were treated conservatively according to the protocol: none required urgent surgery, and they all underwent interval TULAA 8 weeks later. The remaining 196 patients (96.5%) underwent urgent surgery. In 15 out of 196 cases, a primary open access was chosen: in 3 cases for marked abdominal distension, in one case because the surgical team was not sufficiently trained in laparoscopy, and in 11 cases because of palpation of a mass at the induction of anesthesia, and neither surgeons was an expert operator. Sixty-six percent of the primary open accesses were performed in the first two years of the study. Urgent TULAA was carried out in 181 patients. The intraoperative TULAA finding (Figure 2) was uninflamed appendicitis in 18 cases (10%), uncomplicated acute appendicitis (catarrhal/phlegmonous without signs of perforation) in 109 (60%) cases, 49 (27%) cases were either gangrenous or perforated appendicitis with local peritonitis, and 5 (3%) were diffuse peritonitis. The 7 elective cases operated on after antibiotic treatment showed an appendix with adhesions but no acute inflammation. None of these was converted, one required an additional trocar, and no complications were recorded. The mean operatory time for the elective procedure was 43′. 

Of all 181 urgent TULAA, 12 (6.6%) were converted: in 3 cases the intraoperative finding was nonperforated appendicitis with retrocaecal position, in 8 cases there was a perforation with local peritonitis, and one was a diffuse peritonitis. Nine operations were converted by a team of nonexpert surgeons, and 3 by a team in which at least one surgeon was considered expert. Of the 169 nonconverted TULAA procedures, 151 were carried out through the single umbilical port, 16 (9.4%) required a second trocar, and 2 (1.2%) required a third trocar. The mean operative time for single- port TULAA was 52′ (47′ when the first operator was an expert, 55′ when the first was a nonexpert). Among the 181 urgent operations, there were 5 wound infections (3.8%), of which one required a surgical revision, and 5 patients (3.8%) were diagnosed as having postoperative intraperitoneal abscess which were all managed conservatively with intravenous antibiotics.

## 4. Discussion

The TULAA technique was first reported in a large pediatric series by Valla et al. in 1999 [2]. It was described as umbilical one-puncture laparoscopic-assisted appendectomy (UOPLAA), and performed in 200 of preoperatively selected children, that showed no signs of advanced appendicitis or diffuse peritonitis. Our choice of offering TULAA as the first choice operation to the whole spectrum of appendicitis (except local consolidated abscess without fecaliths) was dictated by the fact that this technique can be easily switched to a standard three-port laparoscopic appendectomy, which is widely reported in the literature to be feasible also in advanced form of appendicitis [8]. In our series, only 10% of cases (16 urgent and one elective procedure) required an additional port, and only 2 cases (one perforated appendicitis with local peritonitis and one gangrenous retrocecal appendicitis) required the positioning of 2 additional trocars. The possibility to insert a second or a third trocar in a position that suites the intraoperative findings and the anatomy of the patient, rather than using the standard positions for the traditional laparoscopic procedure, can be of great help during the division of adherences and omentum especially in advanced cases. Similar results in the number of additional ports were reported by Stylianos et al. [9] with 9.8% of 359 cases which required one or two additional ports, by Valla et al. (8%) [2], while Koontz et al. [3] in 2006 reported a lower use of additional trocars in only 2 of 111 patients (2%). The latter report has also a lower rate of conversions (2%) than in our experience and this could be explained by the fact that when TULAA was first introduced in our hospital, the equipment was not well trained in laparoscopy: 75% of our conversions were made by nonexpert members of the staff, and 66% of cases were converted in the first two years of the protocol. This confirms the need of a period of learning curve and the possibility of using this operation as a starting training to acquire laparoscopic abilities. Our operating time (52 minutes) seems longer than other reports: Stylianos et al. 24 minutes [9], Visnjic 33 minutes [10]: these series, however, exclude perforated appendicitis while we include all stages of appendicitis. The only complication we exclude was US confirmed appendiceal abscess with a symptom duration longer than 72 hours, where a conservative management was carried on, according to the current literature [11]. 

Recently, numerous reports appeared in the literature describing the so-called SILS (single-incision laparoscopic surgery) technique where a single umbilical trocar is used to introduce three or four instruments or, as an alternative, at the umbilical site a subcutaneous pocket is created and the natural umbilical fascial defect plus one or two other stab incisions are used to place cannulas (or only instruments) to perform an endocorporeal laparoscopic appendectomy [4, 5, 12]. However, this kind of approach results in longer operating times than standard multiport laparoscopic appendectomy because of the clashing of instruments [12, 13], and it does not have the remarkable reduction in costs that the single trocar operative scope have, compared to standard laparoscopic technique [9, 10].

In our series, 30% of cases were advanced stages of appendicitis but we feel that this is not a condition that should stop from starting the operation with a TULAA approach: the only real contraindication to TULAA is the intestinal loops' huge distension that may exist in some diffuse peritonitis. The concern for umbilical infections due to exteriorization of a suppurative or ruptured appendix can be controlled if adequate skin gauze protection is secured around the umbilical opening when bringing the appendix out. A routine antibiotic prophylaxis is also a recommended procedure before performing an appendectomy [14]. Our rate of wound infections (3.8%) matches perfectly the one calculated for standard three-port laparoscopic appendectomy in a recent meta-analysis comparing open and laparoscopic appendectomy [15], therefore, confirming that the extracorporeal operation does not endanger the umbilical scar.

Petnehazy et al. [16] suggest that TULAA can be a simpler approach for appendectomy in obese children, and even if we did not stratify our population by weight in the present study, a single incision has proved to be a quick and effective approach for this kind of patients also in our hands. 

## 5. Conclusions

According to our experience, TULAA is a safe, minimally invasive approach to patients suffering for acute appendicitis, regardless of the perforation status. It is also a suitable operation for training laparoscopic abilities, and it has low instrumentation requirements. We, therefore, recommend its wide use in the pediatric surgical settings.
